# Socio-Environmental Factors Associated with Self-Rated Oral Health in South Africa: A Multilevel Effects Model

**DOI:** 10.3390/ijerph9103465

**Published:** 2012-10-02

**Authors:** Bukola G. Olutola, Olalekan A. Ayo-Yusuf

**Affiliations:** 1 School of Health Systems and Public Health, University of Pretoria, Pretoria 0002, South Africa; Email: bukola.olutola@gmail.com; 2 Department of Community Dentistry, School of Dentistry, University of Pretoria, Pretoria 0002, South Africa

**Keywords:** self-rated oral health, social capital, mixed-effects model, cell-phone ownership, trust, South Africa

## Abstract

*Aim*: This study examined the influence of the social context in which people live on self-ratings of their oral health. *Method*: This study involved a representative sample of 2,907 South African adults (≥16 years) who participated in the 2007 South African Social Attitude Survey (SASAS). We used the 2005 General Household Survey (n = 107,987 persons from 28,129 households) to obtain living environment characteristics of SASAS participants, including sources of water and energy, and household cell-phone ownership (a proxy measure for the social network available to them). Information obtained from SASAS included socio-demographic data, respondents’ level of trust in people, oral health behaviors and self-rated oral health. *Results*: Of the respondents, 76.3% self-rated their oral health as good. Social context influenced women’s self-rated oral health differently from that of men. Good self-rated oral health was significantly higher among non-smokers, employed respondents and women living in areas with higher household cell-phone ownership. Furthermore, trust and higher social position were associated with good self-rated oral health among men and women respectively. Overall, 55.1% and 18.3% of the variance in self-rated oral health were explained by factors operating at the individual and community levels respectively. *Conclusion*: The findings highlight the potential role of social capital in improving the population’s oral health.

## 1. Introduction

Self-rated health refers to the perception an individual has about his/her health. This perception has been directly associated with the individual’s experiencing physical symptoms [1]. Self-rated oral health is commonly regarded as a global rating of oral health-related quality of life; as it takes into account both the physical and psychological dimensions of health [2]. In particular, people use discomfort in the mouth, the inability of the mouth to function properly, as well as the effect of adverse oral health on social interactions in assessing their own oral health. Factors such as higher education [3], current employment [3,4], being of non-African American race [5,6], higher income [4,7], female gender [4,5] and younger age [5] have all been positively associated with good self-rated oral health. Self-rating of oral health as being good has also been positively associated with a higher number of retained teeth and a recent visit to a dental professional [8]. 

In the 2003/2004 South African Demographic and Health Survey (SADHS), 16% of the respondents reported having had problems with their mouths and/or teeth in the six months preceding the survey [9]. Self-reporting of problems with the mouth and/or teeth varied between ethnicities/race groups and provinces [9]. This geographical variation in self-reported oral health outcomes could be related to variations in the cultural beliefs and traditions, education levels, access to and quality of dental services and other socioeconomic characteristics of the individuals and their environment. 

A study by Turrell *et al*. [10] revealed that there was a significant positive association between a neighborhood’s socioeconomic characteristics and self-rated oral health, irrespective of an individual’s socioeconomic status. Variations in self-rated oral health have been attributed to differences in many socioeconomic and demographic factors which may be observed at both the level of the individual and the neighborhood or community [10]. Most of the prior studies, conducted mostly in developed countries, have focused on individual-level socioeconomic status as determinants of oral health. 

Thus far, only limited information is available on the influence of neighborhood socioeconomic characteristics on the oral health of South Africans, even though addressing socioeconomic disparities in health resulting from decades of apartheid rule [11] remains a public health priority in South Africa. The aim of the present study was therefore to examine the influence of the social context in which people lived on their self-rating of their oral health, independent of personal risk indicators for good oral health. 

## 2. Materials and Methods

### 2.1. Data Source

The individual-level characteristics such as self-rated oral health, oral health behaviors and the socio-demographic characteristics of participants in this study were obtained from the 2007 South African Social Attitudes Survey (SASAS), while the area-level characteristics, outlined further on in this paper were obtained from the General Household Survey (GHS) conducted in 2005. The master samples of both the SASAS and GHS datasets consist of enumeration areas, which are the smallest geographical units that make up municipalities in South Africa. The municipalities (the equivalent of US counties) are the lowest level of government administration and service delivery, hence, they are likely to have meaning for and be significant in terms of where the study participants reside with regard to potential interventions that can be focused on environmental factors that may influence their oral health. Therefore, the two datasets were linked at the municipality level through similar codes that were uniquely assigned to each municipality in the two datasets. A new dataset was then created to form the basis of analysis in the current study. 

The 2007 South African Social Attitudes Survey (SASAS) was a nationally representative sample of adults 16 years and older. Participants were selected following a multi-stage probability sampling strategy. A sample was drawn from the master sample of the South African Human Sciences Research Council (HSRC), which consisted of 1,050 enumeration areas drawn from the 2001 Census. From each of the enumeration areas, 10 visiting points were randomly selected, resulting in a total of 10,500 visiting points in the master sample. The enumeration areas were stratified to be representative of the socio-demographic domains of the province(s), geographical sub-types (tribal areas, formal rural, formal and informal urban) and the four population groups—black, white, coloured (of mixed race) and Indian/Asian—in South Africa [12]. However, for the 2007 SASAS, a total of 4,000 households/visiting points were randomly selected from the master sample. Each person was then randomly selected from each household, without replacement. Efforts were made to secure an interview with each selected person by making three visits before registering the person as non-responding. A sampling weight which took into account the response patterns was applied to produce a representative sample of South Africans ≥ 16 years. 

In the 2005 GHS, multi-stage stratified samples were drawn from Statistics South Africa’s master samples, which, like the SASAS samples, were taken from the enumeration areas established during the 2001 Census 2001 for the 2005 GHS. The detailed methods used in ensuring standardized data collection, interviews and consent procedures for the GHS have been previously published [13]. 

The response rate for the 2007 SASAS was 72.6% (n = 2,907). For the 2005 GHS, the response rate was 87.5% of the targeted 32,146 households (n = 107,987 individuals). The very large sample for the GHS thus provided a unique opportunity to compute area-level characteristics for the corresponding municipalities where the participants of the 2007 SASAS lived (average n = 121 households per municipality). However, two of the municipalities from the 2007 SASAS could not be merged with the 2005 GHS data, because there were no corresponding municipalities in the 2005 GHS. This reduced the sample size by 4%, giving rise to n = 2,791. 

### 2.2. Dependent Variable

Self-rated oral health was assessed by the question “How would you rate your oral health?” and respondents were asked to pick one of the following options: “very good”, “good”, “neither very good nor good”, “poor” and “very poor”. Following the approach used in similar studies [5,10], the options were dichotomized into very good/good (good) and others (neither good nor poor/poor/very poor). 

### 2.3. Independent Variables

#### 2.3.1. Individual-Level Characteristics

The participants indicated their education level by selecting one of several options, with regard to the highest grade completed. The education level was then categorized as: (1) None (no education), (2) Grades 1–7, (3) Grades 8–12, or (4) Higher than Grade 12. 

The respondents were also asked about their current employment status by asking them to pick one of several options. The options were then collapsed into three categories, namely: (1) Employed, (2) Unemployed, and (3) Permanently sick/student/pensioners/housewife not looking for job. 

Subjective socioeconomic status or social position was assessed on a continuous scale using responses to the following question asked in the 2007 SASAS (as in previous studies [14,15]): “In our society there are groups which tend to be towards the top and groups which tend to be towards the bottom. Where would you put yourself on a scale of 1 to 10, where 10 is the top and 1 the bottom?” 

Participants were asked to indicate if they had used dental services in the past year. Considering that utilization of dental services and self-rated oral health could be influenced by the presence of physical symptoms, participants’ recent history of oral health problems was assessed by asking them whether if, within the previous month, they had experienced any of the following common oral conditions: (1) bleeding gums when brushing (symptoms indicative of gum disease), (2) teeth sensitive to heat or cold, (3) bad breath, or (4) none. 

Participants were also asked how frequently they brush and to indicate if they use additional oral hygiene aids such as flossing and mouthwash. Current smokers and snuff users were those who indicated using the respective products “Every day” or “Some days”. 

Similar to the approach used in a previous study [16], trust was measured by asking respondents the extent to which they believed people could be trusted (trust is a proxy measure for social capital.). 

#### 2.3.2. Area-Level Characteristics

Using the 2005 GHS data, area-level social network potential was derived from responses to the question “Is there a cellular telephone available to this household for regular use?” The response was either “Yes” or “No”. The aggregate percentage of cell-phone availability per household or the ‘cell phone network density’ of each municipal area was calculated and assigned to the respective municipal area where respondents to the 2007 SASAS resided. 

Also, using the 2005 GHS, the households that indicated that they did not consult with a health worker were asked why they did not consult any health worker during the past month, and five options were provided, including the option “not necessary”. For this analysis, the responses were dichotomized into two groups of respondents, namely, those who indicated they had experienced some or other barrier to accessing health services (1), and those who indicated experiencing no barrier (0). The proportion of those who had experienced a barrier in accessing health services were calculated for each municipal area as a proxy measure for the level of relative ease of access to health services among those living in that municipality. 

The participants were asked about the household’s main source of water and the respondents had to choose one of many options. Their responses were dichotomized into piped and non-piped water sources, because piped water in South Africa has a relatively lower fluoride level (no public water fluoridation) than non-piped water sources, such as ground water [17]. The proportion of households without piped water was then computed for each area. 

A question about the main source of energy/fuel for the household was also asked. Like the other questions, it had many options which were collapsed into two: (1) those whose main energy source was electricity, and (2) those whose main energy source was not electricity. The proportion of households whose source of energy was not electricity was then computed for each area. 

For the purpose of analysis, each of these municipal or community-level variable values were auto ranked into three categories, namely those lower than the 33.3th percentile, those within the 33.3th to 66.7th percentiles and higher than the 66.7th percentile. 

### 2.4. Data Analyses

The data was analyzed using STATA version 10 (STATA Corp Inc., College Station, TX, USA). Group differences were tested using chi-squares and the t-test for categorical and continuous variables respectively. All statistical tests were two-tailed and the level of significance was set at *p* < 0.05. A multilevel binomial logit link model was used to assess the effect of community-level factors on self-rated oral health after the individual-level factors had been controlled for [18]. The main outcome variable was good self-rated oral health, and three sequential models were generated [19]. Model 1 was the empty model, which contained only the outcome variable with no independent variable. Area-level variables were added to Model 1 to become Model 2, and then the individual-level variables were added to Model 2 to become Model 3, which was the final model. 

The variance in self-rated oral health at the community level was noted for each of the models and the changes in these variance estimates were recorded as the model was built from an empty model to sequentially include the area-level factors and individual-level factors. These changes were noted to denote the level of contribution made by each set of factors/variables toward explaining variations in self-rated oral health across municipalities. The criterion for inclusion of the variables into the logistic model from the bivariate analysis was set at *p* < 0.25, while the decisive criterion for retention in the model was *p* < 0.05 [20]. 

Following a suggestion by Hosmer and Lemeshow [20], factors that did not meet the *p* < 0.25 criterion were finally introduced into the model to identify factors that were not significantly related to self-rated good oral health in themselves, but that made an important contribution in the presence of other variables. The log-likelihood ratio test (LR-test) was then used to examine whether or not the multilevel/random effect model was significantly better than an ordinary logistic regression model. Considering the previously noted modifying effect of gender on the observed association between level of education and self-rated oral health [9], the interactions between gender and education as well as between gender and social positioning as a measure of socioeconomic status were explored. As a result, additional analyses were carried out separately for male and female respondents. 

The interaction between gender and indicators of social capital was also explored based on the findings from other studies showing gender differences in the relationship between health and social capital [21,22]. 

## 3. Results and Discussion

### 3.1. Results

The average age of the study participants was 36.9 (SD = 0.6) years. Of the respondents, 76.3% (n = 2,067) reported good oral health, 51.7% (n = 1,617) were female and 77.1% (n = 1,755) identified themselves as black Africans. The unemployment rate among the studied participants varied significantly by race, with black Africans having the highest unemployment rate. A significantly greater proportion of respondents in the age group between 16 and 24 years than respondents who were 65 years and older rated their oral health as good (Table 1). Those who reported dental attendance in the past year were less likely to have rated their oral health as good than those who did not. Other socio-demographic and behavioral factors associated with self-rated good oral health are shown in Table 1. 

**Table 1:** Bivariate relationship between self-rated good oral health status and individual-level risk factors.

	Characteristics		Self-rated good oral health % (n)	*p*-value
**Socio-demographic factors **				
	Gender			0.00
		Male	80.6 (939)	
		Female	71.8 (1,189)	
	Ethnicity			0.03
		Black	75.6 (1,315)	
		Coloured	69.5 (278)	
		Indian or Asian	87.7 (273)	
		White	81.5 (262)	
	Age (Years)			0.00
		16-24	89.8 (604)	
		25-34	84.6 (523)	
		35-44	77.3 (474)	
		45-54	65.7 (270)	
		55-64	53.4 (154)	
		>65	38.1 (96)	
	Education			0.00
		>Grade 12	90.3 (297)	
		Grade 12	89.2 (663)	
		<Grade 12	71.5 (1,101)	
		None	30.9 (61)	
	Employment status			0.00
		Employed	85.3 (838)	
		Housewives/students/Pensioners	81.9 (542)	
		Unemployed	66.1 (739)	
	Resident			0.01
		Urban	79.4 (1,476)	
		Rural	70.0 (591)	
**Social capital proxy measure**				
	Trust in people			0.46
		Not trusted	75.4 (1,419)	
		Trusted	77.6 (708)	
**Tobacco use**				
	Currently smoking			0.00
		No	78.3 (1,675)	
		Yes	68.6 (433)	
	Currently using snuff			0.00
		No	77.8 (2,048)	
		Yes	47.6 (60)	
**Oral health status and behaviour**				
	Past year attendance for dental care			0.03
		No	78.5 (1,301)	
		Yes	71.8 (823)	
**Recent history of oral health problems**				
	Tooth sensitivity to heat or cold			0.00
		No	79.9 (1,851)	
		Yes	56.9 (277)	
	Bleeding gums when brushing			0.00
		No	80.5 (1,859)	
		Yes	57.4 (269)	
	Bad breath			0.00
		No	78.5 (1,996)	
		Yes	55.4 (132)	
**Oral hygiene practice**				
	Toothbrushing frequency			0.00
		No brushing	51.7 (85)	
		Brushed not everyday	52.9 (120)	
		Brushed at least once daily	74.8 (753)	
		Brushed at least twice daily	85.0 (1,109)	
	Daily use of mouthwash			0.26
		No	75.7 (1,870)	
		Yes	80.1 (258)	
	Flossing at least twice a week			0.13
		No	75.8 (2,019)	
		Yes	84.1 (109)	
	Used toothpicks at least twice a week			0.30
		No	75.8 (1,998)	
		Yes	81.2 (109)	

In general, 61.9% of households reported having a cell-phone and 12.1% reported having no tap water (Table 2). Compared to those who rated their oral health as poor, a higher proportion of those who rated their oral health as good lived in areas with a significantly higher proportion of households with a cell-phone (58.7% *vs.* 62.9%; *p* < 0.01). Self-rated good oral health was also more common among those who lived in areas with fewer households using the public health facilities and areas with fewer households that did not have access to basic infrastructure such as piped water or electricity (Table 2). 

**Table 2:** Descriptive characteristics of community-level variables by self-rated good oral health.

Area-level characteristics %	Total Mean % (SE)	Self-rated oral health status	Area-level Mean % (SE) *	*p*-value for differences in area-level mean %
Households with cell phone	61.9 (2.0)			0.00
		Poor	58.7 (1.6)	
		Good	62.9 (2.2)	
Residents using public health facilities	59.5 (2.6)			0.00
		Poor	62.6 (2.4)	
		Good	58.6 (2.8)	
Households without piped water	12.1 (2.3)			0.01
		Poor	16.2 (2.9)	
		Good	11.0 (2.2)	
Households that had experienced a barrier in contacting a health worker	25.9 (3.0)			0.01
		Poor	29.4 (2.8)	
		Good	24.8 (3.1)	
% Households whose main source of energy was not electricity	19.8 (1.9)			0.02
		Poor	22.5 (2.1)	
		Good	19.0 (1.9)	

* SE = standard error.

It was found that in a multivariate adjusted model only cell-phone ownership remained significantly associated with self-rated good oral health. The adjusted odds ratio (AOR) was 1.74 (95% CI; 1.16–2.61) in communities with the highest proportion of households with cell phones (*i.e.*, the highest cell-phone density), when compared with those living in areas with the lowest proportion of households with cell-phones (Table 3). After controlling for individual-level risk factors, respondents living in areas with an intermediate proportion of households with cell-phones were those most likely to have self-rated good oral health. The effect of the highest cell-phone density was attenuated by the inclusion of individuals’ socioeconomic circumstances. It is pertinent to note that 55.1% of the total variance in self-rated good oral health was explained by the individual-level factors, while only 18.3% of the variance was explained by the community-level characteristics. 

**Table 3:** Association of self-rated good oral health with individual and community-level characteristics determined by multilevel logistic regression.

		Model 1 (Null model)	Model 2	Model 3
**Random effects**				
	Area-level variance (SE)	0.60 (0.14)	0.49 (0.13)	0.22 (0.09)
**Fixed effects**				
*Area-level characteristics*				
Household Cell-phone ownership	Lowest		1.0 (Reference category)	1.0 (Reference category)
	Intermediate		1.53 (1.04–2.25)	1.60 (1.12–2.27)
	Highest		1.74 (1.16–2.61)	1.48 (1.02–2.15)
*Individual-level variables*				
Gender				
	Male			1.0 (Reference category)
	Female			0.61 (0.48–0.78)
Age				
	16–24			1.0 (Reference category)
	25–34			0.67 (0.46–0.97)
	35–44			0.48 (0.33–0.69)
	45–54			0.24 (0.16–0.34)
	55–64			0.16 (0.11–0.24)
	>65			0.12 (0.08–0.19)
Education				
	>Grade 12			1.0 (Reference category)
	Grade 12			0.89 (0.57–1.41)
	<Grade 12			0.55 (0.36–0.85)
	None			0.30 (0.17–0.54)
Employment status				
	Employed			1.0 (Reference category)
	Unemployed			0.58 (0.44–0.76)
	Housewives/students/Pensioners			0.69 (0.50–0.96)
Subjective socio-economic status				1.10 (1.03–1.17)
Trust in people				
	Not trusted			1.0 (Reference category)
	Trusted			1.32(1.04–1.67)
Smoking status/currently smoking				
	No			1.0 (Reference category)
	Yes			0.41 (0.31–0.53)
Past use of dental services				
	No			1.0 (Reference category)
	Yes			0.59 (0.47–0.74)
Frequency of tooth-brushing				
	No brushing			1.0 (Reference category)
	Brushed not everyday			1.74 (1.01–3.01)
	Brushed at least once daily			2.90 (1.86–4.54)
	Brushed at least twice daily			3.87 (2.47–6.06)
Mouthwash				
	No			1.0 (Reference category)
	Yes			2.33 (1.52–3.57)
Tooth sensitivity				
	No			1.0 (Reference category)
	Yes			0.54 (0.42–0.71)
Bleeding gums				
	No			1.0 (Reference category)
	Yes			0.39 (0.30–0.51)
Bad breath				
	No			1.0 (Reference category)
	Yes			0.60 (0.42–0.86)
–2 Log-likelihood		3,021.7	3,013.8	2,275.2
*P*-value		0.00	0.00	0.00

The significant positive association between higher education and self-rated good oral health remained clear, with those with no education being 70% less likely to report good oral health than those with a Grade 12 or higher level of education (AOR = 0.30; 95% CI; 0.17–0.54) (Table 3). There was a significant interaction between gender and trust (*p* < 0.01), as well as between gender and education (*p* = 0.05); therefore the analyses were stratified by gender (Table 4 and Table 5). Unlike women, men who reported that people could be trusted were more likely to report self-rated good oral health than those who said people could not be trusted (AOR = 1.91; 95% CI; 1.29–2.83). Furthermore, unlike women, no statistically significant association was found among men between self-rated oral health and any of the area-level factors (Table 4). Moreover, in contrast to women, relative social positioning within society was not significantly associated with men’s self-rating of their oral health. The social gradient with regard to the level of education and employment among men (Table 4) was steeper than that observed among women (Table 5). 

**Table 4:** Multilevel model of determinants of self-rated good oral health among men.

Characteristics		AOR (95% Conf. Interval)
Age	16–24	1.0 (Reference category)
	25–34	0.57 (0.29–1.11)
	35–44	0.45 (0.23–0.89)
	45–54	0.18 (0.09–0.35)
	55–64	0.16 (0.08–0.32)
	>65	0.17 (0.08–0.36)
Education	>Grade 12	1.0 (Reference category)
	Grade 12	1.21 (0.62–2.37)
	<Grade 12	0.53 (0.30–0.96)
	None	0.15 (0.07–0.35)
Employment	Employed	1.0 (Reference category)
	Unemployed	0.44 (0.29–0.66)
	Housewives/students/pensioners	0.69 (0.34–1.39)
Trust in People	Not trusted	1.0 (Reference category)
	Trusted	1.91 (1.29–2.83)
Currently smoking	No	1.0 (Reference category)
	Yes	0.37 (0.25–0.53)
Past use of dental services	No	1.0 (Reference category)
	Yes	0.53 (0.36–0.77)
Frequency of tooth-brushing	No brushing	1.0 (Reference category)
	Brushed not everyday	1.83 (0.82–4.10)
	Brushed at least once daily	3.16 (1.63–6.12)
	Brushed at least twice daily	4.12 (2.10–8.06)
Bad breath	No	1.0 (Reference category)
	Yes	0.56 (0.34–0.94)
Bleeding gum	No	1.0 (Reference category)
	Yes	0.34 (0.22–0.53)

Irrespective of gender, self-rated good oral health was less likely among those who reported frequently bleeding gums, tooth sensitivity and bad breath. Furthermore, non-smokers and those who reported frequent tooth-brushing were significantly more likely to have self-rated their oral health as good than smokers and those who do not brush daily or who brushed less frequently respectively. 

**Table 5:** Multilevel model of determinants of self-rated good oral health among women.

Characteristics		AOR (95% Conf. Interval)
Households with Cell–phone	Area with the lowest proportion	1.0 (Reference category)
	Intermediate	1.96 (1.28–3.01)
	High	1.74 (1.11–2.74)
Age	16–24	1.0 (Reference category)
	25–34	0.68 (0.42–1.09)
	35–44	0.46 (0.29–0.74)
	45–54	0.26 (0.16–0.42)
	55–64	0.15 (0.09–0.26)
	>65	0.10 (0.05–0.17)
Education	>Grade 12	1.0 (Reference category)
	Grade 12	0.64 (0.33–1.24)
	<Grade 12	0.44 (0.23–0.83)
	None	0.35 (0.15–0.79)
Employment	Employed	1.0 (Reference category)
	Unemployed	0.68 (0.47–0.98)
	Housewives/students/pensioners	0.65 (0.44–0.98)
Subjective Social position rank		1.17 (1.08–1.27)
Currently smoking	No	1.0 (Reference category)
	Yes	0.40 (0.27–0.59)
Past use of dental services	No	1.0 (Reference category)
	Yes	0.61 (0.45–0.81)
Tooth sensitivity	No	1.0 (Reference category)
	Yes	0.48 (0.34–0.67)
Bad breath	No	1.0 (Reference category)
	Yes	0.56 (0.34–0.92)
Bleeding gums	No	1.0 (Reference category)
	Yes	0.40 (0.28–0.57)
Mouth wash	No	1.0 (Reference category)
	Yes	2.63 (1.51–4.60)
Frequency of tooth–brushing	No brushing	1.0 (Reference category)
	Brushed not everyday	1.67 (0.80–3.48)
	Brushed at least once daily	2.62 (1.45–4.75)
	Brushed at least twice daily	3.60 (1.99–6.50)

AOR—Adjusted odds ratio.

### 3.2. Discussion

In addition to exploring personal risk factors, this study examined the influence of the social context in which people lived on their self-ratings of their oral health. This study found a positive association between area-level and individual-level social capital and self-rated good oral health, after controlling for potential confounders at the individual level. In particular, male participants who trust people and women who live in areas with high household cell phone ownership were more likely to rate their oral health as good, independent of the level of any other social disadvantage. However, there was no evidence of a significant independent association with the other area-level factors explored in this study. 

In general, living in an area with high cell phone ownership/network density increases the odds that a person will rate his/her oral health as good. A previous study has suggested that the use of cell phones and other mobile technologies are important in developing, strengthening and maintaining friendships, and they also affect relationships with family members [23]. Thus, cell phone ownership, which may reflect the level of infrastructural development in an area, may also represent a component of social capital, which has previously been shown to be positively associated with better self-rated oral health [24]. Cell phones are used for social interaction [25], and socially connected individuals may have greater social support, which may in turn lower the risk of psychological stress among people [26]. Psychosocial stress has in turn been demonstrated to be a significant determinant of poor oral health [27] and is well-represented in Locker’s model of oral health [28]. 

However, none of the area-level variables, including cell-phone ownership, were significant in men. This suggests that unlike women, the social context in which men live does not influence their self-ratings of oral health as much as it influences those of women. Individual level social capital, namely trust, was significant only among men, thus supporting previous reports that men are more individualistic and more engaged in formal collaborations [29] and not as community-oriented and informal in their associations as women [29,30]. Women belong to more informal groups, which allow them to form stronger kinship and friendship relations [31], which can be used in influencing health-related behaviors [32]. It is more likely that oral functional limitation or impairment that compromises social functioning will have a greater impact on women’s health-related quality of life. 

The observation that area-level cell-phone ownership was relevant only for women’s oral health may also be related to the fact that in South Africa, more women than men own cell phones [33] and that women are more likely to use the cell phones mainly for social reasons, such as maintaining informal relationships, and also to contact family or friends in case of an emergency. By contrast, men use cell phones more for work-related issues such as maintaining employment or professional contacts, which are forms of formal relationships, than for social reasons [33]. 

This study, like other studies, has shown that a greater proportion of males than females rated their oral health as good [34,35]. This observation may be related to the fact that, on the one hand, women have been found to be more likely to report oral and facial symptoms than men [36] and, on the other hand, it has been shown in this study and others [36,37] that people often rate their oral health as poor because they display oral symptoms. The observed gender differences in self-rated oral health may also be related to the fact that women have been shown to be more self-critical [38]. Gender differences in self-rated oral health may also reflect the fact that women in South Africa have a significantly lower disposable income and level of education [39] and thus have less access to oral health care. 

Consistent with previous studies’ findings [6,10,40], both higher education and employment status, which were the objective measures of the individuals’ socio-economic status, were related to self-rating oral health as good. It has been suggested that subjective social status, which is an important correlate of health, captures the dimensions of social status that the indicators of objective socio-economic status cannot [41]. Increased subjective social status has been associated with reduced levels of psychological distress, which in turn has a positive effect on health [14]. It was therefore not surprising that respondents had better self-rated oral health for every increment in social position ranking. However, it should be noted that the effect of subjective social status on self-rated oral health in this study was particularly relevant only for women. Indeed, women (unlike men) have been suggested to take into account both emotional stress and physical health when rating their health [42]. Therefore, a woman who scored herself higher on the subjective social status gradient would perhaps be more likely to have less emotional stress and would be more likely to rate her oral health as good than another woman who rated herself lower on the social status gradient. 

The elimination of the initially observed racial differences in self-rated oral health after controlling for individual-level and area-level socio-economic factors suggests that racial differences in self-rated oral health are mediated by differences in individual socioeconomic circumstances and the social context in which different races live in South Africa. Indeed, whites and Indians, who constituted the highest proportion of those with self-rated good health, often reside in urban areas [43] with better health and social infrastructure (including cell-phone networks) and employment opportunities, and therefore have more access to social capital and dental services than members of other race groups. 

Interestingly, those who have made use of dental services in the past were less likely to rate their oral health as good. It has been previously reported [44] that the most common reason for dental visits in South Africa is to address particular symptoms. Indeed, preventive dental visits have been associated with a self-rating of oral health as good, while restorative or symptomatic visits have been associated with a self-rating of oral health as poor [45,46]. Thus, consistent with a previous study’s findings [34], those who reported oral health problems such as tooth sensitivity, bleeding gums and bad breath were significantly less likely to self-rate their oral health as good. Similarly, smokers who tend to develop halitosis, tooth-staining and suppressed gingivitis [47] and are less likely to make use of dental services for routine care [48,49] were also found to be less likely to self-rate their oral health as good. In line with a previous study’s findings, there was a positive association between optimal oral hygiene practices such as brushing at least twice daily and the use of mouthwash and good self-reported oral health [35]. 

One of the limitations of this study was that it was cross-sectional, which precluded clear evidence on causality, given the limited information on the temporal order of events. However, the inclusion of the 2005 GHS data provided some information on a temporal order of events for area-level factors. Admittedly, some people living in the respective areas during the 2007 SASAS may well not have resided in the same area in 2005. It is, however, unlikely that the proportion of people who moved out of the municipalities has changed so significantly over the two-year period as to significantly change the findings in this study. 

Using self-rated oral health over a clinical measurement may have introduced reporting bias, but it has been argued that self-reported health status is a better determinant of demand for care or services than professional and clinical diagnosis; so the use of this patient-centered measure may inform service demand and thus service planning better [50]. 

It is pertinent that there was still a large unexplained residual random effect after controlling for potential confounders. The residual effect could be a result of some as yet unmeasured factors, such as the level of fluoride in the water in the different areas, which has been shown to influence the prevalence of dental caries [51]—a condition that may affect respondents’ self-rating of their oral health status. Finally, the area-level measures had different variances which might have influenced their level of relative contribution in a multivariate analysis. Despite this study’s limitations, this study has produced useful information that has the potential to influence policy and practice and to inform further studies on the social and environmental determinants of oral health. 

The strengths of this study lie in the use of large, nationally representative datasets and the use of a statistical approach that allowed for the separation of composition influences from contextual influences on health outcomes. 

## 4. Conclusions

This study’s findings are consistent with the social health framework that suggests that self-rated health is a combination of considerations of physical oral functioning and of social relationships [52]. In particular, this study’s findings highlight the role of a hitherto understudied, but growing, community resource, namely cell phones in low- and middle-income countries, which was shown in this study to attenuate the impact of socioeconomic disadvantages on oral health. 

## References

[1] Bailis D.S., Segall A., Chipperfield J.G. (2003). Two views of self-rated general health status. Soc. Sci. Med..

[2] Gift H.C., Atchison K.A., Dayton C.M. (1997). Conceptualizing oral health and oral health-related quality of life. Soc. Sci. Med..

[3] Gilbert L., Soskolne V. (2003). Self-assessed health—A case study of social differentials in Soweto, South Africa. Health Place.

[4] Finlayson T.L., Williams D.R., Siefert K., Jackson J.S., Nowjack-Raymer R. (2010). Oral health disparities and psychosocial correlates of self-rated oral health in the National Survey of American life. Am. J. Public Health.

[5] Pattusi M.P., Peres K.G., Boing A.F., Peres M.A., Da Costa J.S.D. (2010). Self-rated oral health and associated factors in Brazilian elders. Comm. Dent. Oral Epidemiol..

[6] Gilbert L. (1994). Social factors and self-assessed oral health in South Africa. Comm. Dent. Oral Epidemiol..

[7] Coulter I., Yamamoto J.M., Marcus M., Freed J., Der-Martirosian C., Guzman-Becerra N., Brown L.J., Guay A. (2004). Self-reported oral health of enrollees in capitated and fee-for-service dental benefit plans. J. Am. Dent. Assoc..

[8] Jones J.A., Kressin N.R., Spiro A., Randall C.W., Miller D.R., Hayes C., Kazis L., Garcia R.I. (2001). Self-reported and clinical oral health in users of VA health care. J. Gerontol. A Biol. Sci. Med. Sci..

[9] South African Department of Health (2003). South African Demographic and Health Survey (SADHS). http://www.info.gov.za/view/DownloadFileAction?id=90140.

[10] Turrell G., Sanders A.E., Slade G.D., Spencer A.J., Marcenes W. (2007). The independent contribution of neighbourhood disadvantage and individual-level socio-economic position to self-reported oral health: A multilevel analysis. Comm. Dent. Oral Epidemiol..

[11] Coovadia H., Jewkes R., Barron P., Sanders D., McIntyre D. (2009). The health and health system of South Africa: Historical roots of current public health challenges. Lancet.

[12] Statistics South Africa Census 2001: Metadata. http://www.statssa.gov.za/census01/html/C2001metadate.asp.

[13] (2005). General Household Survey. http://www.statssa.gov.za/publications/P0318/P0318July2005.

[14] Operario D., Adler N.E., Williams D.R. (2004). Subjective social status: Reliability and predictive utility for global health. Psychol. Health.

[15] Adler N.E., Epel E., Castellazzo G., Ickovics J. (2000). Relationship of subjective and objective social status with psychological and physical health: Preliminary data in healthy white women. Health Psychol..

[16] Kim D., Baum C.F., Ganz M.L., Subramanian S.V., Kawachi I. (2011). The contextual effects of social capital on health: A cross-national instrumental variable analysis. Soc. Sci. Med..

[17] Muller W.J., Heath R.G.M., Villet M.H. (1998). Finding the optimum: Fluoridation of portable water in South Africa. Water SA.

[18] Diez-Roux A.V. (2002). A glossary for multilevel analysis. J. Epidemiol. Community Health.

[19] Subramanian S.V., Kawachi I., Kennedy B.P. (2001). Does the state you live in make a difference? Multilevel analysis of self-rated health in the U.S. Soc. Sci. Med..

[20] Hosmer D.W., Lemeshow S. (2000). Applied Logistic Regression.

[21] Locher J., Ritchie C., Roth D., Baker P., Bodner E., Allman R. (2005). Social isolation, support, and capital and nutritional risk in an older sample: Ethnic and gender differences. Soc. Sci. Med..

[22] Kavanagh A., Bentley R., Turrell D., Broom D., Subramanian S. (2006). Does gender modify associations between self-rated health and the social and economic characteristics of local environments?. J. Epidemiol. Community Health.

[23] Yang S., Kurnia S., Lee H., Kim S. The Impact of Mobile Phone Use on Social Capital Development: A Preliminary Study in South Korea. http://www.pacis-net.org/file/2008/PACIS2008_Camera-Ready_Paper_138.pdf.

[24] Furuta M., Ekuni D., Takao S., Suzuki E., Morita M., Kawachi I. (2011). Social capital and self-rated oral health among young people. Comm. Dent. Oral Epidemiol..

[25] Scott N., Batchelor S., Ridley J., Jorgensen B. The Impact of Mobile Phones in Africa. Prepared for the Commission for Africa. http://gamos.org.uk/couksite/Projects/Docs/Mobile%20phones%20in%20Africa/Full%20Report.pdf.

[26] Phongsavan P., Chey T., Bauman A., Brooks R., Silove D. (2006). Social capital, socio-economic status and psychological distress among Australian adults. Soc. Sci. Med..

[27] Sanders A.E., Spencer A.J. (2005). Why do poor adults rate their oral health poorly?. Aust. Dent. J..

[28] Locker D. (1988). Measuring oral health: A conceptual framework. Commu. Dent. Health.

[29] Molyneux M. (2002). Gender and the silence of social capital: Lessons from Latin America. Dev. Change.

[30] Norris P., Inglehart R. Gendering Social Capital. Proceedings of the Conference on Gender and Social Capital.

[31] Westermann O., Ashby J., Pretty J. (2005). Gender and social capital: The importance of gender differences for the maturity and effectiveness of natural resource management groups. World Dev..

[32] Pattussi M.P. Neighbourhood social capital and oral health in adolescents.

[33] Gillward A., Milek A., Stork C. (2010). Towards Evidence-Based ICT Policy and Regulation. Gender Assessment of ICT Access and Usage in Africa. http://www.ictworks.org/sites/default/files/uploaded_pics/2009/Gender_Paper_Sept_2010.pdf.

[34] Chen M.S., Hunter P. (1996). Oral health and quality of life in New Zealand: A social perspective. Soc. Sci. Med..

[35] Locker D., Jokovic A., Payne B. (1997). Life circumstances, lifestyles and oral health among older Canadians. Comm. Dent. Health.

[36] Locker D., Miller Y. (1994). Subjectively reported oral health status in an adult population. Comm. Dent. Oral Epidemiol..

[37] Newton J.T., Corrigan M., Gibbons D.E., Locker D. (2003). The self-assessed oral health status of individuals from White, Indian, Chinese and Black Caribbean communities in South-east England. Comm. Dent. Oral Epidemiol..

[38] Tada A., Hanada N. (2004). Sexual differences in oral health behaviour and factors associated with oral health behaviour in Japanese young adults. Public Health.

[39] Walker L., Gilbert L. (2002). HIV/AIDS: South African women at risk. AJAR.

[40] Matthias R.E., Atchison K.A., Lubbin J.E., De Jong F., Schweitzer S.O. (1995). Factors affecting self-ratings of oral health. J. Public Health Dent..

[41] Demakakos P., Nazroo J., Breeze E., Marmot M. (2008). Socioeconomic status and health: The role of subjective social status. Soc. Sci. Med..

[42] Benyamini Y., Leventhal E.A., Leventhal H. (2000). Gender differences in processing information for making self-assessments of health. Psychosom. Med..

[43] Charasse-Pouele C., Fournier M. (2006). Health disparities between racial groups in South Africa: A decomposition analysis. Soc. Sci. Med..

[44] Mickenautsch S., Frencken J.E., Van’t Hof M.A. (2007). Atraumatic restorative treatment and dental anxiety in outpatients attending public oral health clinics in South Africa. J. Public Health Dent..

[45] Maupome G., Peters D., White A. (2004). Use of clinical services compared with patients’ perceptions of and satisfaction with oral health status. J. Public Health Dent..

[46] Afonso-Souza G., Nadanovsky P., Chor D., Faerstein E., Werneck G.L., Lopes C.S. (2007). Association between routine visits for dental checkup and self-perceived oral health in an adult population in Rio de Janeiro: The Pro-Saude study. Comm. Dent. Oral Epidemiol..

[47] Johnson N.W. (2004). The role of the dental team in tobacco cessation. Eur. J. Dent. Educ..

[48] Wu B. (2007). Dental service utilization among urban and rural older adults in China—A brief communication. J. Public Health Dent..

[49] Kounari-Koletsi H., Tzavara C., Tountas Y. (2011). Health-related lifestyle behaviours, socio-demographic characteristics and use of dental health services in Greek adults. Comm. Dent. Health.

[50] Locker D. (1996). Application of self-reported assessments of oral health outcomes. J. Dent. Educ..

[51] Postma T.C., Ayo-Yusuf O.A., Van Wyk P.J. (2008). Socio-demographic correlates of early childhood caries prevalence and severity in a developing country—South Africa. Int. Dent. J..

[52] Hahn E.A., DeVellis R.F., Bode R.K., Garcia S.F., Castel L.D., Eisen S.V., Bosworth H.B., Heinemann A.W., Rothrock N., Cella B. (2010). Measuring social health in the patient-reported outcomes measurement information system (PROMIS): Item bank development and testing. Qual. Life Res..

